# Neurovascular coupling in bone regeneration

**DOI:** 10.1038/s12276-022-00899-6

**Published:** 2022-11-29

**Authors:** Qizhi Qin, Seungyong Lee, Nirali Patel, Kalah Walden, Mario Gomez-Salazar, Benjamin Levi, Aaron W. James

**Affiliations:** 1 Department of Pathology, Johns Hopkins University School of Medicine, Baltimore, MD 21205 USA; 2 Department of Physiology, College of Graduate Studies, Midwestern University, Glendale, AZ 85308 USA; 3 Department of Physical Education, Incheon National University, Incheon, 22012 South Korea; 4 Arizona College of Osteopathic Medicine, Midwestern University, Glendale, AZ 85308 USA; 5 Departments of Surgery, UT Southwestern Medical Center, Dallas, TX 75390 USA

**Keywords:** Bone, Neuro-vascular interactions

## Abstract

The mammalian skeletal system is densely innervated by both neural and vascular networks. Peripheral nerves in the skeleton include sensory and sympathetic nerves. The crosstalk between skeletal and neural tissues is critical for skeletal development and regeneration. The cellular processes of osteogenesis and angiogenesis are coupled in both physiological and pathophysiological contexts. The cellular and molecular regulation of osteogenesis and angiogenesis have yet to be fully defined. This review will provide a detailed characterization of the regulatory role of nerves and blood vessels during bone regeneration. Furthermore, given the importance of the spatial relationship between nerves and blood vessels in bone, we discuss neurovascular coupling during physiological and pathological bone formation. A better understanding of the interactions between nerves and blood vessels will inform future novel therapeutic neural and vascular targeting for clinical bone repair and regeneration.

## Bone regeneration: Interplay of nerve and blood vessel growth

Further exploration of the interactions between nerve cells and vascularization (blood vessel development into tissue) during bone repair could provide therapeutic targets for bone diseases, including osteoarthritis and bone cancer. Seungyoung Lee at Incheon National University (Incheon, South Korea), Aaron James at Johns Hopkins School of Medicine (Baltimore, USA), and co-workers reviewed the roles of peripheral nerve cell groups and blood vessels in the regulation of bone remodeling. The growth of peripheral nerves and blood vessels is coordinated and functionally interdependent during skeletal development and repair. The density of nerve growth increases during bone remodeling, closely followed by increased vascularization. Inhibiting nerve signaling delays healing following a bone fracture. Two families of growth factor proteins, one expressed by sensory nerves and the other involved in triggering vascularization, could play key roles in this interaction between nerves and blood vessels.

## Introduction

Bone tissue is vital for all mammalian species as a living organ system that allows body structure and movement. Bone remodeling maintains bone strength and mineral-calcium homeostasis, and it involves bone cells, including bone-resorbing osteoclasts, bone-forming osteoblasts, osteocytes, and bone-lining cells^1,2^. The skeleton has a rich innervation of both sensory and sympathetic nerve fibers in conjunction with the bone vascular system^3–5^.

Although it has only recently begun to be in the spotlight, the regulatory role of the skeletal, nervous system, including sensory, sympathetic, and parasympathetic nerves, has been examined for many years^3–9^. Each peripheral nerve fiber subtype has distinct functions within bone^10–12^. In addition to performing the general roles of nerve fibers, such as providing motor control of muscle fibers, delivering sensory information from the periphery to the central nervous system (CNS), and regulating autonomic functions^13^, skeletal nerve fibers also provide paracrine stimuli, such as neuropeptide Y (NPY), leptin, calcitonin gene-related peptide (CGRP), and substance P (SP)^3,14–16^. Moreover, adrenergic receptors and other receptors for neuropeptides residing on osteoblasts and osteoclasts express axon guidance molecules such as semaphorins, netrins, and neurotrophins, which further potentiate nerve ingrowth^17,18^. Neurotrophins promote the survival of neurons and target innervation by activating the receptors TrkA and p75^19^. Semaphorins serve as axon guidance molecules during nervous system development^20^. The growth of peripheral nerve fibers and the initial innervation and neurotransmission into the bone tissue regulate bone remodeling and metabolism^21^. Furthermore, nerve ingrowth is an essential upstream mediator of endochondral ossification during bone growth^22^ and intramembranous bone formation during growth and repair^7,8,23^. These accumulating study findings suggest that the stimuli provided by peripheral afferent neurons represent an essential requirement for proper skeletal growth, homeostasis, and repair^7,10,11^.

Recent studies suggest that the bone vascular system plays a significant role in bone remodeling. Bone marrow arteries and capillaries are essential components of bone multicellular units^24–27^. The bone vasculature is also important for bone marrow homeostasis^28,29^. Declines in vascular function and perfusion are predicted to cause reduced bone volume, bone density, osteoblast activity, and increased osteoclastic resorption^30–32^. Accumulating evidence suggests that the skeletal, nervous, and vascular systems influence one another. Recent works have documented that the majority of sympathetic and parasympathetic nerves are located in association with blood vessels^10,33^. Moreover, nerves accompanying blood vessels innervate the primary and secondary ossification center during embryonic skeletal development, whereby CGRP-expressed sensory nerves are detected near osteoblasts and the area of high osteogenic activity^34,35^. However, the extent to which nerve-vessel coupling contributes to improvements in skeletal remodeling and metabolism remains an intriguing unanswered question. As such, this review presents evidence of the roles of neurovascular coupling in bone regeneration, as well as the clinical entity of heterotopic ossification (HO), and summarizes the potential clinical implications of neurovascular coupling in bone tissue.

## Nerve and vascular distribution within the bone

The skeleton is densely innervated by sensory and sympathetic nerves. Peptidergic sensory nerve fibers are prevalent in the skeletal system of vertebrate species and express the neuropeptides CGRP and SP^3,36^. The general role of sensory nerves is to transmit internal and external information from the periphery to the CNS for processing of sensory information. The autonomic nervous system (ANS), including sympathetic and parasympathetic nerves, is a part of the efferent peripheral nervous system (PNS) that innervates and regulates all organs and involuntary functions in the body. Sympathetic nerves are abundant in bone and express tyrosine hydroxylase (TH)^10,36,37^. Both sensory and autonomic nerve fibers in bone are closely linked to bone metabolism and bone remodeling cellular activities.

Although sensory and autonomic nerve fibers have been shown to have mixed effects, both types of nerve fibers are strongly associated with blood vessels during skeletal development and bone repair. During development, blood vessels run into the medullary cavity with their accompanying nerves along the shaft within the bone^38,39^, and the collection of sinusoidal vessels returns to the central nutrient vein along with nerves^38,40^. In cortical bone, sensory and sympathetic nerve fibers are largely confined to vascularized Haversian canals^41^. Moreover, CGRP^+^ and NF200^+^ sensory nerve fibers are associated with blood vessels located in the Haversian canals, and TH^+^ sympathetic nerve fibers are observed in the bone marrow and wrapping around blood vessels^42^. In our prior observations, *Thy1*-YFP pan-neuronal reporter activity closely paralleled CD31^+^ blood vessels during calvarial defect healing^8^. A similar nerve-vessel coupling was observed by Chartier et al., in which CGRP^+^ sensory nerves and TH^+^ sympathetic nerves were colocalized with CD31^+^ blood vessels in the periosteum^41^. Moreover, neuropeptide Y (NPY) fibers were identified around cerebral blood vessels^43^ and in the medullary cavity accompanying blood vessels in the long bones^11^.

In summary, the skeleton is covered by both neural and vascular networks. Their intimate spatial relationship suggests that they modulate each other. However, the interaction between the nerves and vessels remains largely unknown, which has been a trending topic of research in recent years.

## Neurovascular coupling during bone repair and regeneration

### Nerve signals during fracture repair and regeneration processes

Primary sensory and sympathetic axons covering the periosteal bone are required for bone tissue repair and fracture healing by rearranging and reinnervating the nerve fibers into injury sites^3,44^. The nerve growth factor (NGF) is the most important neurotrophin and neuropeptide involved in the regulation of the growth, maintenance, proliferation, and survival of sensory neurons^45^. Tropomyosin receptor kinase A (TrkA) is the high-affinity neurotrophin receptor for NGF, and TrkA^+^ sensory neurons are extensively innervated in the periosteum^42,46^. A previous study in mouse long bone stress fracture repair showed that NGF expression within a nascent callus was considerably upregulated at 3 days and up to 7 days postfracture with a concomitant increase in axonal ingrowth and sprouting, followed by vascularization and ossification^7^. Consistent with this study, our recent work demonstrated that during calvarial bone defect healing, neurotrophic NGF and TrkA signaling play a crucial role in normal calvarial bone regeneration^8^. Moreover, Tomlinson et al. reported that communications between osteoblasts and sensory nerves via NGF-TrkA signaling are required for mechanical loading-induced bone formation^47^. In contrast, studies in murine injury models have revealed that surgical and chemical denervation of sensory nerves resulted in impaired fracture healing and decreased bone formation^48–51^. In sensory nerves, the disruption of TrkA catalytic activity by the small molecule 1NMPP1 impairs the sequential events of long bone fracture and flat bone defect healing. This disruption also leads to reduced osteoblast activity and abrogated blood vessels in the defect area^7,8^. In addition to NGF-TrkA signaling, common sensory nerves that express CGRP and SP have been shown to play a role in rat tibial fracture healing, whereby sensory nerve innervation was present on the periosteal layers and influenced bone regeneration by stimulating newly produced bony calli^52,53^. The regulatory role of sensory nerves in bone formation was also observed in both mammalian models and human patients with nerve dysfunction. Moreover, reduced peripheral innervation was associated with delayed fracture healing^54^, and the loss of the sensory nerves led to a reduction in new bone quality in rabbits^55^.

In regard to the ANS contribution to the bone healing process, in contrast to the sensory nerves, NPY-positive autonomic nerves were found to be decreased at the fracture or defect regions at early time points, while the presence of NPY-expressing nerve ingrowth and sprouting through Y1 receptor-associated signaling was observed. This accelerated the late phase of bone formation by increasing the osteogenic differentiation of mesenchymal stem cells (MSCs)^56–58^. A recent finding has demonstrated that the disruption of sympathetic nerve stimulation via sympathectomy hinders motor function and locomotion and delays fracture callus formation in the late phase of the bone healing process^59^. Although the relationship between the nervous system and the cells that drive bone formation is not fully understood, it is known that neurotransmitters can affect osteoblast formation^60,61^. In addition, osteoblasts are regulated by peripheral nerve-derived norepinephrine^62,63^, and bone contributes to the completeness of the microenvironment by accommodating nerves in the bones to provide bone stability^13^. Recently, our group used spatial transcriptomics of the developing cranium to reveal the importance of nerve signaling in the regulation of bone patterning and the differentiation of bone precursors, highlighting the important role of neural signaling in tissue regulation^23^.

Given our data and previously reported outcomes demonstrating the essential role of NGF-TrkA signaling, CGRP- and SP-associated sensory nerves, and autonomic nerve fibers in bone repair and remodeling, both sensory and autonomic innervation are employed for stimulating vascular ingrowth and sprouting, increasing osteogenic differentiation, and facilitating the delivery of osteogenic factors to the fracture site.

### Vascular signals during bone repair and regeneration processes

Crucial roles of the vascular system for bone and bone marrow function, such as bone homeostasis and hematopoietic activity, have also been suggested. Bone blood vessels deliver O_2_, nutrients, and systemic hormones to bone and bone marrow, and they remove waste products. Bone blood vessels also transport immune and precursor cells to and from bone and bone marrow and are fundamental components of the bone multicellular unit^24,25,64^. In addition, blood vessels enriching bone plays a significant role in hematopoietic stem cell (HSC) niches, which are essential for bone homeostasis^29^. It is presumed that the dysfunction of bone and bone marrow is highly associated with dysfunction in the skeletal vascular system^31,32,65^. For example, declines in bone mass were found to be associated with diminished bone blood flow and impaired vasodilator function of bone blood vessels^66–68^ in rats and humans.

Normally, skeletal blood vessel sprouting and vascular function-related substances, such as nitric oxide, are augmented quickly following fracture incidence due to rapid increases in blood flow to the fracture region^69^. Blood supply is a challenge for bone repair and regeneration. To achieve proper bone repair, the local microcirculation must be intact. Many factors stimulate angiogenesis, some of which include fibroblast growth factors, transforming growth factors, platelet-derived growth factors, and vascular endothelial growth factors (VEGF)^70,71^. VEGF has been shown to increase angiogenesis, which increases vascular permeability and the recruitment of MSCs and osteoprogenitor cells. VEGF has the ability to attract MSCs and promote differentiation into osteogenic cell types. Studies have shown that when VEGF is inhibited, bone formation and invasion of the vasculature are decreased^71,72^. When the VEGF receptor is antagonized, this inhibits bone regeneration by bone morphogenetic protein (BMP) 4 and 2, as osteogenesis is induced by these osteogenic cytokines. Kaigler et al. showed that when VEGF is inhibited, angiogenesis and osteogenesis are inhibited^73^. However, new vascularization and bone regeneration occurred with the controlled release of VEGF. In addition, BMPs act synergistically with VEGF to enhance cell survival, cartilage formation, resorption, bone formation, and mineralization^71,72^. BMPs and VEGF synergistically enhance angiogenesis, osteogenic genes, and MSC responses to BMP6, while the ratio of VEGF/BMP is an important mediator. A high ratio of VEGF/BMP2 was found to produce well-formed mineralized bone but a decreased amount of bone compared to a low ratio of VEGF/BMP2^22^.

### Neurovascular crosstalk and potential sources of bone repair and regeneration

Neurovascular coupling is a combination of vascular smooth muscle cells, neurons, and astrocyte glial cells. Both neurons and astrocytes respond to increases in vasoactive metabolites that adjust blood flow. Glutamate released from presynaptic neurons in the presence of oxygen stimulates the activation of nitric oxide synthase (NOS), which produces NO to act directly as a dilator on parenchymal arterioles^74^. Astrocytes respond to glutamate in the presence of oxygen to activate a cascading pathway to produce arachidonic acid, which leads to the production of epoxyeicosatrienoic acid and prostaglandins^75^. By having dual stimuli, there is an even greater effect on the increase in blood flow^76^. The neurovascular theory for nerves in bone emerged in the 1980s following the observation that blood flow was increased in the joints of patients with diabetic neuropathy^77^. In addition, the crucial functions of both blood vessels and nerve stimuli in hematopoietic niches have been described. Nociceptive nerves are required to induce HSC mobilization, while sympathetic and sensory nerves collaboratively maintain HSC homeostasis in the bone marrow^78,79^.

After a bone is fractured, growth factors and cytokines recruit osteoprogenitor cells to the affected site^80,81^. As the bone remodels, angiogenesis allows for an invasion of vascular networks into the bone to restore normal circulation to remove/replace the necrotic bone tissue^82^, and nerve fibers are closely associated with blood vessels^7,22^. Our results showed that nerve ingrowth occurred before revascularization and contributed to vascular regrowth^8^. Moreover, impaired nerve reinnervation is accompanied by decreased vascularity and reduced osteogenic capacity, which delayed bone defect healing^8^. Single-cell RNA sequencing data from a trauma-induced HO model revealed that NGF was highly expressed in perivascular stem cells and that NGF-TrkA signaling increased angiogenesis and vascular regeneration^9,83–85^. The inhibition of TrkA signaling resulted in delayed vascular invasion in primary and secondary ossification centers, which further reduced the length and volume of the femur in a rodent model^22^. We also demonstrated that chemical genetic inhibition of TrkA signaling led to blunted sensory nerve reinnervation and diminished revascularization, thereby impairing fracture healing^7^.

VEGF is one of the primary mediators promoting angiogenesis while binding to VEGF receptors 1 and 2 promotes bone repair and has direct effects on the nervous systems in terms of axon branching and survival^86^. Conversely, NGF exhibits angiogenic properties^87^. The injection of NGF directly into the site of the defect enhances vascularization and osteogenesis^84^. SP could promote angiogenesis and osteogenic differentiation by upregulating VEGF expression and enhancing BMP2 signaling^61,88,89^. In addition, cholinergic neurons release the neuropeptide vasoactive intestinal peptide, which improves cranial defect healing and bone formation by increasing VEGF expression in MSCs and stimulating angiogenesis^90^.

Clinically, vascularization and neurotization are important in the care of bone trauma, tumors, and infection. A study by Fan et al. showed how tissue-engineered bone (TEB) is affected by implanted vessels and nerves by using a mouse model where the femur was exposed, and osteotomy was performed^91^. Bone tissue gradually increased in TEB along with the femoral blood vessels and saphenous nerve implantation at the defect site^91^. However, new bone formation was highest following saphenous nerve implantation, whereas vascular implantation led to more and better-quality blood vessels^91^. Interestingly, vascular implantation led to increased CGRP and NPY expression, which was presumably associated with the nerves in the walls of the blood vessels, which in turn stimulated neurotization and osteoblast activity and inhibited osteoclasts^91^. Moreover, the levels of CGRP and NPY were found at the highest levels with saphenous nerve implantation, which led to better neurotization of the TEB and more blood vessels^91^. Taken together, these findings demonstrate that the increase in vasculature was due to the capillary network within the nerve fibers, which led to an increase in osteogenesis.

Given the roles of nerve-vascular interactions and functions in bone repair and regeneration, therapeutic targeting of neural and vascular signals may be a promising therapeutic option for bone regeneration.

## The role of neurovascular coupling in ectopic bone formation

### Nerve signals during the ectopic bone formation process

In the context of HO, the observation of a coregulation of nerves and bone implicates nerves as a critical mediator in HO pathogenesis. HO has been frequently observed in those who have paroxysmal sympathetic hyperactivity^92,93^. Moreover, the incidence of HO is dramatically increased in patients with traumatic CNS injury^94^. This evidence suggests an association between HO and peripheral nerves. Peripheral nerves enable HO through the release of molecules such as SP and the induction of neuroinflammation^95^. In addition, Nguyen et al. reported BMP2 as a dual-function cytokine that promotes HO via osteoinductive action and neuroinflammation induction^96^. Salisbury et al. demonstrated that sensory nerves contribute to HO development and that the inhibition of sensory nerves led to reduced HO formation by suppressing BMP2-mediated SP and CGRP^97^. Altogether, these findings suggest that HO formation depends on peripheral innervation.

In addition to the nociceptor potential of sensory nerves, a large body of literature has demonstrated the role of sensory nerves in osteoanabolic potential, as well as in regulating bone regeneration and formation. In the context of HO, our recent studies demonstrated that vascular smooth muscle cell- and pericyte-induced NGF-mediated axon innervation accompanied posttraumatic HO in a murine traumatic HO model^9^. Notably, in this study, surgical denervation blunted axonal invasion and reduced chondrogenesis and ectopic bone formation. Similarly, either NGF deletion or TrkA inhibition phenocopied impaired axonal ingrowth and heterotopic bone formation.

### Vascular signals during the ectopic bone formation process

It has long been known that angiogenesis and vascularization make a remarkable contribution to ectopic bone formation. The representative angiogenic factor VEGFA, for instance, influences vascularity in the form of ectopic bone formation^98,99^. Although this is not fully understood, the activity of a tissue ischemia marker, hypoxia-inducible factor (HIF), induced by a local hypoxic microenvironment, initiates the pathogenesis of HO^100,101^, which induces the expression of VEGF, thereby enhancing angiogenesis, cartilage differentiation, and ultimately HO formation^99,102,103^. Furthermore, vascular histomorphometric analysis in human HO samples demonstrated that the pathophysiological processes of osteogenesis and angiogenesis are coupled in a time- and space-dependent manner^104^. Macrophage- and brown adipocyte-derived VEGF promoted the occurrence and pathogenesis of HO^99,105^. More recently, by using a murine posttraumatic HO model, our group demonstrated that traumatic HO is highly vascular and identified the critical role of HIF1α in traumatic HO^102^. Moreover, the release of VEGFA from mesenchymal cells drives HO formation^106^. The effects of angiogenic factors can be recapitulated in vivo with a transgenic mouse model of HO. Dilling et al. reported an increase in endothelial cells and a corresponding increase in *VEGF* mRNA expression in the area of HO, which preceded the inception of mesenchymal contribution and cartilaginous tissue formation^99^. This suggests that vascularization plays a pivotal role in MSC condensation and chondrogenesis during HO progression.

### Neurovascular crosstalk and potential sources of HO progression

Although neurovascular coupling is well demonstrated during skeletal development, until recently, it has remained largely unknown in the context of HO. Our studies revealed that experimental models of HO are hyperinnervated, and sensory nerve innervation is an early and necessary feature for HO^9^. Furthermore, by using the same posttraumatic HO model, our group identified neural-derived angiogenic factors and demonstrated a unifying mechanism for neurovascular crosstalk in the context of HO^106^. Hwang et al. demonstrated that HO lesions are highly vascularized and that mesenchymal-derived VEGFA drives ectopic bone formation^106^. These findings implicate the interaction between nerves and blood vessels during HO formation. More recently, by using the traumatic HO model, we demonstrated that progressive neurovascular infiltration accompanies posttraumatic HO^107^. Qin et al. demonstrated progressive ingrowth in overall Tubb3^+^ nerve fibers and CD31^+^ blood vessels by 1883% and 3468%, respectively, over the early period (up to 3 weeks) of HO progression. These findings suggest that increased nerve fiber innervation mirrors the upregulation of endothelial vascularity during the early phase of HO development^107^. The authors further investigated neurovascular coupling by using sciatic neurectomy, which greatly inhibited axonal ingrowth and reduced vascular ingrowth into the injury site. This observation was phenocopied by independent mechanisms, and chemical or genetic inhibition of axonal ingrowth led to similar deficits in injury site angiogenesis, suggesting that neuronal perturbation impairs nerve and vessel infiltration and potential neurovascular crosstalk^107^. Single-cell transcriptomic analysis identified that vascular and perivascular cell clusters were present at the injury site, whereas surgical denervation led to a reduction in the proliferative index and a reduction in overall VEGF signaling activity in vascular and perivascular cell clusters^107^. Furthermore, a combination of single-cell transcriptomic approaches within the dorsal root ganglia identified key neural-derived angiogenic paracrine factors that may mediate neurovascular coupling^107^.

Reciprocally, the potential mutual impact of vessels on nerves during HO was investigated by the same group. By using a previously reported animal model, vascular ingrowth and HO were inhibited by conditional deletion of *Vegfa* in *Prrx1*-Cre animals^106^. The deletion of *Vegfa* had significant effects on vascular density, as expected. In addition, a significant reduction in TUBB3^+^ axons was also found within the HO site (unpublished data). Taken together, these findings demonstrate that it is plausible to assert that nerves and blood vessels are closely interrelated and promote ectopic bone formation interdependently.

## Summary

Nerve and blood vessel ingrowth are spatially coordinated and functionally interdependent during skeletal development and regeneration. The mechanisms of neurovascular coupling are complex, and a variety of cells and molecules participate in neurovascular crosstalk. Many regulatory effects remain unknown. In this review, we discussed the role of nerves and blood vessels in the regulation of bone remodeling and repair and explored the interaction between nerves and vessels during physiological and pathological scenarios. Nerves densely innervate bone, and increased nerve density coincides with bone remodeling and regeneration^11^. Moreover, nerve innervation is normally followed by vascularization during bone fracture repair. In contrast, the inhibition of nerve signaling is accompanied by blunted revascularization and delayed fracture healing^7^. The secretion of VEGF by sensory nerves is the major factor influencing vascular growth and differentiation^108^. NGF-TrkA signaling promotes vascularization by inducing sensory innervation during embryonic development^22^. Furthermore, NGF leads to endothelial cell proliferation and inflammation^109^. NGF-expressing cells may play a key role in neurovascular coupling, which remains to be further investigated. On the other hand, VEGF promotes reinnervation and stimulates axonal outgrowth in the peripheral nervous system^110^. In this regard, further research on the neurotrophic activity of VEGF in bone remodeling and fracture healing is needed.

A better understanding of neurovascular coupling and underlying cellular and molecular drivers could advance the identification of novel targets for the treatment and clinical prevention of diseases such as heterotopic bone formation, osteoarthritis, and even bone tumors. Research focusing on neurovascular coupling during bone regeneration could also contribute to the development of novel differentiation factors and biomaterials for bone repair.
